# Implementation of whole genome sequencing for tuberculosis diagnostics in a low-middle income, high MDR-TB burden country

**DOI:** 10.1038/s41598-021-94297-z

**Published:** 2021-07-28

**Authors:** Monica Vogel, Christian Utpatel, Caroline Corbett, Thomas A. Kohl, Altyn Iskakova, Sevim Ahmedov, Uladzimir Antonenka, Viola Dreyer, Ainura Ibrahimova, Chynara Kamarli, Dilorom Kosimova, Vanessa Mohr, Evgeni Sahalchyk, Meerim Sydykova, Nagira Umetalieva, Abdylat Kadyrov, Gulmira Kalmambetova, Stefan Niemann, Harald Hoffmann

**Affiliations:** 1 Institute of Microbiology and Laboratory Medicine, Department IML Red GmbH, WHO - Supranational Tuberculosis Reference Laboratory Munich-Gauting, Robert Koch-Allee 2, 82131 Gauting, Germany; 2 Molecular and Experimental Mycobacteriology, Research Center Borstel, Borstel, Germany; 3 German Center for Infection Research, Partner Site Hamburg-Lübeck-Borstel-Riems, Borstel, Germany; 4 Republican Tuberculosis Reference Laboratory, Bishkek, Kyrgyz Republic; 5 USAID, Bureau for Global Health, TB Division, Washington, DC USA; 6 ABT Associates, Defeat TB Project Management, Bishkek, Kyrgyz Republic; 7 USAID, Country Office, Bishkek, Kyrgyz Republic; 8 Republican Tuberculosis Center, National Tuberculosis Project Management, Bishkek, Kyrgyz Republic; 9 SYNLAB Gauting, SYNLAB Human Genetics, Munich-Gauting, Germany

**Keywords:** Next-generation sequencing, Tuberculosis, Molecular biology, Microbiology, Infectious-disease diagnostics

## Abstract

Whole genome sequencing (WGS) is revolutionary for diagnostics of TB and its mutations associated with drug-resistances, but its uptake in low- and middle-income countries is hindered by concerns of implementation feasibility. Here, we provide a proof of concept for its successful implementation in such a setting. WGS was implemented in the Kyrgyz Republic. We estimated needs of up to 55 TB-WGS per week and chose the MiSeq platform (Illumina, USA) because of its capacity of up to 60 TB-WGS per week. The project’s timeline was completed in 93-weeks. Costs of large equipment and accompanying costs were 222,065 USD and 8462 USD, respectively. The first 174 WGS costed 277 USD per sequence, but this was skewed by training inefficiencies. Based on real prices and presuming optimal utilization of WGS capacities, WGS costs could drop to 167 and 141 USD per WGS using MiSeq Reagent Kits v2 (500-cycles) and v3 (600-cycles), respectively. Five trainings were required to prepare the staff for autonomous WGS which cost 48,250 USD. External assessment confirmed excellent performance of WGS by the Kyrgyz laboratory in an interlaboratory comparison of 30 *M. tuberculosis* genomes showing complete agreeance of results.

## Introduction

The World Health Organization (WHO) aims to reduce worldwide tuberculosis (TB) incidence by 90% until 2035^1^. Currently, TB incidence is falling at a rate of 2% per year, impeded mainly by the uncontrolled pandemic of drug-resistant TB (DR-TB). Of particular concern are multi-drug resistant (MDR) (defined as resistance toward isoniazid [INH] and rifampicin [RIF]) *Mycobacterium tuberculosis* complex (*Mtbc*) strains^2^. Regimens informed by drug susceptibility testing (DST) assure optimal results^3^; however, world-wide MDR-TB treatment outcomes are poor with cure rates below 60%^4^. A major reason for this is the lack of comprehensive DST in most high MDR-TB burden countries, leading to unsatisfactory anti-TB activity of the applied regimens^3,5,6^. In the past few years the emerging next generation sequencing technologies of whole genome sequencing (WGS) and targeted sequencing (tgNGS) have shown great potential for fast and reliable determination of TB resistance patterns with genomic DST (gDST)^5,7–10^.Although there are clear benefits to incorporating gDST into diagnostics^10^, WHO has indicated that “the uptake of those technologies for DR-TB diagnosis is hindered by concerns regarding costs, integration into laboratory workflows, technical training and skill requirements of utilization, and the need of expert guidance regarding the management and clinical interpretation of sequencing data”^11^. The current cost estimates of $28.21 USD to $69.44 USD per WGS of *Mtbc* isolates were developed for the US market and might not apply to high-incidence settings^12^. Furthermore, incorporating WGS into the national diagnostic algorithms involves intensive training and programmatic planning which is challenged by a lack of advanced molecular biological education^13,14^. These perceived barriers need to be overcome in high-prevalence countries where the added benefits of gDST could make significant progress towards decreasing MDR-TB incidence^15^.

Here we report on the successful implementation and validation of WGS in the Kyrgyz Republic, supported by USAID and national partners. Our experience provides valuable insights on the real and perceived implementation costs and challenges, and could be a resource for scientists and TB Programs who aim to implement WGS in high-incidence, high MDR-TB burden, low- to middle-income settings. We will present details on key elements regarding (i) infrastructure, human capacity building, budget planning; and (ii) options for external quality control of gDST in high-prevalence countries.

## Methods

The Kyrgyz Republic (population: 6.3 M) is a land-locked Central Asian Republic the world-bank ranks as lower-middle income country (GDP per capita 1277 USD p.a.)^16^. In 2017 when the project started, TB incidence was 144/100,000 and 7695 TB cases were notified^17^. Of those, 3151 had bacteriologically confirmed pulmonary TB; an estimated 2300 were MDR/RR-TB (1372 bacteriologically confirmed); 134 were confirmed XDR-TB. In 2018, the National Reference Laboratory (NRL) had performed 1191 pDSTs from positive cultures of MDR/RR-TB-cases (*unpublished Kyrgyz TB Program data*).

The requirements of the WGS platform which would best suit the needs of the Kyrgyz TB program were calculated using the epidemiological figures outlined above. The complete resistance patterns of all 1372 confirmed MDR/RR-TB cases would have to be determined per year to integrate WGS into the national TB diagnostic algorithm. When accounting for the possibility to expand WGS to 10 to 20% of bacteriologically confirmed non-MDR/RR pulmonary TB cases for surveillance, the resulting calculated WGS capacity is up to 2900 per year corresponding to 55 sequences per week. We have thus chosen the MiSeq platform with a capacity of up to 30 *Mtbc* genomes per run and two runs per week. Accompanying larger equipment is listed in Table 1, and includes a workstation for bioinformatic analyses fulfilling at least the specifications of Table S1. The equipment was installed in a room dedicated only to WGS, with approximately 650 × 5000 mm bench space, no through traffic, heating, ventilation, and air conditioning (HVAC), dust filters, sunlight protection, and limited entry for authorized personnel only.

**Table 1:** Large equipment and kits with associated manufacturer information.

Category	Item (model/brand) additional cost	Manufacturer	Distributor	Catalogue Number	Units	Net price per unit (USD)	Net sub-Total (USD)
Large equipment	Next generation sequencer (MiSeq system V2)	Illumina	Alliance Global	SY-410-1003	1	118,800.00	118,800.00
	– Ultimate 2-year Warranty	Illumina	Alliance Global	IL-SV-420-1008	1	26,039.65	26,039.65
	– Installation and initial instruction	Illumina			1	10,1900.00	11,900.00
	Fluorometer/Spectrophotometer (DS-11FX + Microvolume)	Denovix	BioLabtech,	9027304040	1	12,165.80	12,165.80
	Fragment analyzing system^a^ (Advanced Analytical 5200 FA System)	Agilent	Vizamed	FSv2-CE2F	1	47,880.00	47,880.00
	– Installation and initial instruction	Agilent	Vizamed		1	2933.22	2933.22
	Computer^b^	Locally built-to-order	Locally built-to-order		1	1646.96	1646.96
	Uninterrupted Power Supply APC/BR1500GI UPS Por/AVR/1 500 VA/865 W	APC	Ermex		1	700.00	700.00
Sub-total					8	222,065.63	222,065.63
Kit and supplement-ary supplies	dsDNA High Sensitivity Kit—1000 assay	Denovix	BioLabtech	BioLabtech	2	395.00	790.00
	dsDNA Broad Range Kit—1000 assay	Denovix	BioLabtech	BioLabtech	2	455.48	910.96
	Denovix vials 0.5 mL thin wall (500 pc. Box)	Denovix	BioLabtech	TUBE-PCR-0.5-500	6	63.00	378.00
	Fragment Analyzer qualification kit	Agilent	Vizamed	DNF-FSEW-OQ	1	900.60	900.60
	Capillary conditioner	Agilent		DNF-475-0100	1	74.10	74.10
	12 capillary array cartridge 50 µm	Agilent	Vizamed	A2300-1250-3355	1	853.00	853.00
	HS NGS Fragment Analysis Kit 1–6000 bp	Agilent	Vizamed	DNF-474-0500	1	2117.00	2117.00
	MiSeq Reagent Kit v2 (500 cycle)	Illumina	Alliance Global	MS-102-200	17	1452.00	24,684.00
	MiSeq Reagent Kit v3 (600 cycle)	Illumina	Alliance Global	MS-102-3003	6	1896.00	11,376.00
	Nextera XT DNA Sample Preparation Kit (96 Samples)	Illumina	Alliance Global	FC-131-1096	4	3924.00	15,696.00
	Nextera XT Index Kit v2 Set A (96 Indices, 384 Samples)	Illumina	Alliance Global	FC-131-2001	2	1224.00	2448.00
	PhiX Control Kit	Illumina	Alliance Global	FC-110-3001	4	192.00	768.00
Sub-total					47		60,995.66
Total					55		283,061.29

^a^Including 1-year warranty.

^b^Computer specification detailed in Supplementary Table S2.

A clinical microbiologist and a laboratory technician of the Kyrgyz NRL were trained as WGS master users. Both specialists had basic knowledge in genetics and more than five-year experience in TB diagnostics and were initially trained for two weeks on the protocols of DNA purification, library preparation, and bioinformatic data analysis at the WHO Supranational Reference Laboratories (SRL) of Gauting and Borstel, Germany. Later-on, on-site trainings were provided by specialists from the German SRL partners and by the distributors of the fragment analyzer and the Illumina MiSseq. All trainings are described in Table S2. Training costs were calculated based on fees of 1000 USD per day for senior experts (professor, medical director), 750 USD for post-docs, and 500 USD for technicians multiplied by the number of training days (Table S2).

Procurement costs were classified in terms of net (item price) and gross per-item costs (item price plus proportionate accompanying costs of shipment, custom clearance and consulting). The first procurement of consumables was sufficient for 2000 DNA extractions and 800 WGS sequences (Tables 1, S3). Real per-sequence costs were calculated after the first 174 WGS by dividing the costs of actually consumed materials by 174. The theoretical long-term per-sequence costs were estimated based on the use of the MiSeq Reagent kits v2 (500-cycles) and v3 (600-cycles) under the assumption they are always completely consumed with the optimal number of samples.

As no official external quality assessment (EQA) is available for WGS of *Mtbc*, we organized it as interlaboratory comparison. Genomic DNA of 30 randomly selected clinical *Mtbc* isolates was extracted at the Kyrgyz NRL using the CTAB protocol (SOP provided in the supplement materials), photometrically checked and separated into two identical 25 µl aliquots. NRL and RCB independently prepared libraries using the Nextera XT kit following the instructions of the manufacturer and using a modified Nextera protocol, respectively^9,18^. Both laboratories independently performed and bioinformatically analyzed WGS of the isolates on MiSeq and NextSeq 500 (Illumina, USA), respectively. Reads from all 60 WGS datasets were analyzed for variant positions using the MTBseq pipeline^19^. Variant positions were defined when the following criteria were met: the variant base was covered by at least four forward and reverse reads with a Phred score of minimum 20; the base differed from the *M. tuberculosis* H37Rv reference allele; the allele was present in a proportion of at least 75%. Variant positions from the 60 datasets were combined when at least 95% of the samples fulfilled the aforementioned thresholds.

For phylogenetic analysis, a concatenated single nucleotide polymorphism (SNP) alignment was created based on variant positions (excluding insertions and deletions, SNPs within a window of 12 bp or located in repetitive regions, and resistance-associated genes). The alignment was used for the calculation of a maximum parsimony tree using BioNumerics version 7.6 (AppliedMaths, Sint-Martens-Latem, Belgium). Datasets preliminarily flagged as discordant by the phylogenetic reconstruction were pairwise analysed for non-matching SNP positions or mixed infections by the presence of signature SNPs for two different TB lineages or alternative allele frequency cluster detection.

Once the EQA was completed a two-day workshop was conducted where national and international TB-experts planned the integration of WGS into the national diagnostic algorithm in the Kyrgyz Republic.

### Ethics approval and consent to participate

The project protocol has been reviewed by the Ethical committee under the Ministry of Health of the Kyrgyz Republic and considered as not requiring ethical approval as neither personal nor clinical data were used nor interventions were performed on humans or animals.

## Results

The entire project occurred within a 93-week period (Fig. S1). All equipment was on site in the Kyrgyz NRL 45 weeks after the procurement process began which was significantly delayed due to unexpectedly long delivery and custom clearance times of key equipment. The implementation of WGS took 50 weeks measured as the time between the first training and the first autonomous WGS.

Net costs of large equipment were equal to 222,065 USD plus accompanying costs of 8,462 USD (3.8%) (Tables 1, S1). Gross material costs incurred for the first 174 genomes were 277.34 USD per WGS and sample. Due to efficiency increasing with practice, long-term sequencing costs were calculated to be 167.33 USD and 141.46 USD per WGS/sample using the MiSeq Reagent Kit v2 (500-cycles) and v3 (600-cycles), respectively (Table 2). Three shipments of reagents each taking approximately two months from order to delivery were necessary for the sequencing of 174 genomes due to the short-guaranteed shelf life of only 3.5 months for the Nexera XT library preparation kits.

**Table 2:** Sequencing and reagent costs per sequence measured during implementation, and calculated costs per sequence using V2-kits or V3-kits once NGS has been established and staff have been properly trained.

Work-step	Measured costs per sequence^a^	Calculated costs per sequence	
		V2-kits^b^	V3-kits^c^
DNA extraction	$ 4.05	$ 4.05	$ 4.05
Denovix DNA Quant + QC	$ 2.88	$ 1.31	$ 1.31
Library preparation	$ 91.25	$ 59.61	$ 59.61
Fragment analysis/library QC	$ 14.7	$ 3.83	$ 3.83
Sequencing	$ 156.32	$ 90.41	$ 64.53
Shipping	$ 8.13	$ 8.13	$ 8.13
Total	$ 277.34	$ 167.33	$ 141.46

^a^Measured for first 174 sequences.

^b^Theoretical costs based on 16 samples/run.

^c^Theoretical costs based on 30 samples/run.

Six training units were required to sufficiently prepare the NRL staff for autonomous WGS and data analysis (Fig. S1; Table S2). Skills were consolidated in a subsequent refresher training. Bioinformatic capacity building covered the identification of resistance associated polymorphisms using PhyResSE, cluster/transmission detection with MTBseq^20^, and phylogenetic reconstruction and visualization with BioNumerics (AppliedMaths, Sint-Martens-Latem, Belgium). All trainings together caused calculated costs of 48,250 USD.

**Figure 1:**
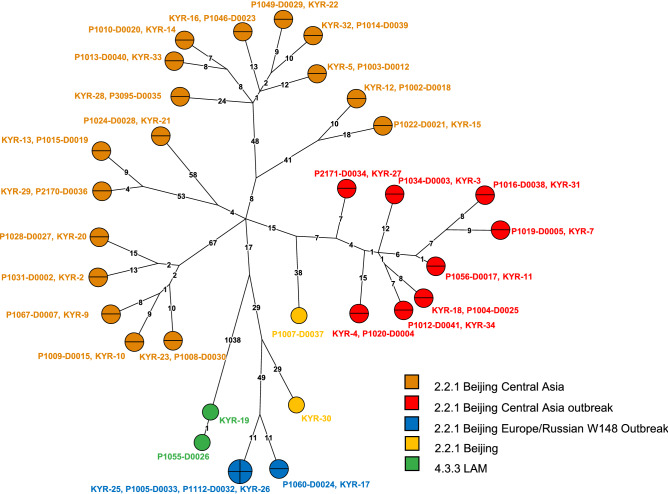
External quality control by interlaboratory comparison of 30 pairs of WGS of random clinical *Mtbc* isolates independently produced by Kyrgyz NRL and RCB. Genotypes are indicated for the sake of completeness according to Merker et al.^31^. For 28 analyzed datasets, the calculated pairwise distances were zero. Two samples yielded divergent pairs of results. The DNA sample pair KYR-19/P1055-D0026 (green dots) showed a wild-type and a SNP population each of approximately 50% at the divergent position (further details see Fig. S2). KYR-30 and P1007-D0037 (yellow dots) were derived from a mixed infection with two *Mtbc* strains of Beijing 2.2.1 lineage. The presence of two alternative allele clusters at a frequency of approximately 40–60% resulted in either the wildtype or alternative alleles being identified and reported by NRL and RCB at the variant positions.

As control of success and for an external quality assessment, 60 sequencing datasets originating from the parallel WGS of 30 random clinical *Mtbc* isolates at NRL and RCB were evaluated for their quality based on reached coverage and percentage of covered genome from MTBseq analyses. All datasets produced at the NRL reached a mean genome coverage depth of at least 50-fold and an unambiguous coverage breadth of 95% (proportion of the reference genome covered by at least four forward and reverse reads, 75% allele frequency and four reads of main allele with a Phred score of at least 20) indicating high quality eligible for variant analysis. For 28 out of the 30 analyzed dataset pairs the calculated pairwise distances were zero and no difference in phylogenetic informative SNP positions could be observed between datasets originating from the same DNA sample (Fig. 1). One of the two divergent samples showed a wild-type and an SNP population each of approximately 50% at the divergent position. Due to the inherent technical variation in library preparation and sequencing technology, the SNP was sequenced with a frequency of 52% at the RCB which reported the allele, but only of 43% at the NRL which reported the wildtype (Fig. S2). When the samples were pairwise analyzed without the background of the other 58 sample pairs and the variant positions introduced by them into the concatenated SNP alignment, the zero SNP difference was verified. For the second divergent sample pair a difference of more than 100 SNPs was identified due to a mixed infection with two *Mtbc* strains of the Beijing 2.2.1 lineage. The presence of two alternative allele clusters at a frequency of approximately 40–60% resulted in either the wildtype or alternative alleles being identified and reported.

**Figure 2:**
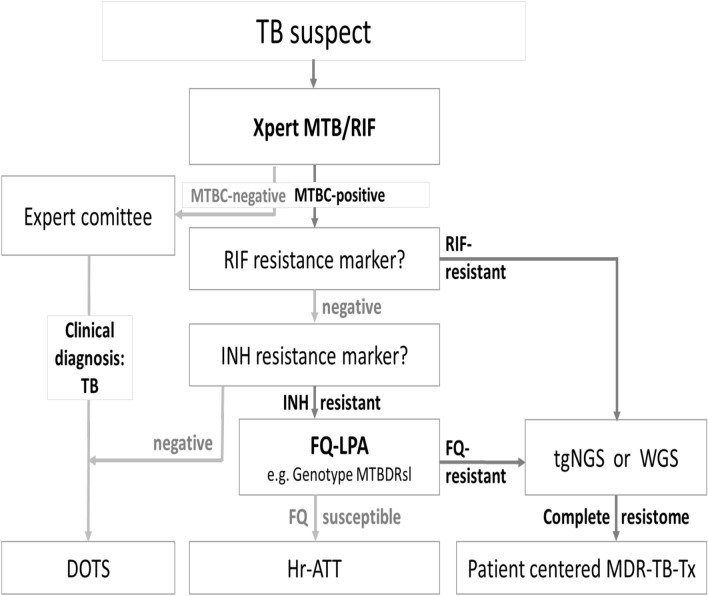
Updated diagnostic algorithm including NGS. In a workshop with national and international TB stakeholders of Kyrgyzstan, the diagnostic algorithm of TB was complemented with NGS as new diagnostic tool. According to this new consensus algorithm, NGS shall be applied to all rifampicin resistant and to all isoniazid and fluoroquinolone poly-drug-resistant cases. *RIF* rifampicin; *INH* isoniazid; *FQ* fluoroquinolone; *LPA* line probe assay; *tgNGS* targeted next generation sequencing; *WGS* whole genome sequencing; *DOTS* directly observed therapy short course; *Hr-ATT* anti-tuberculosis therapy for isoniazid resistant TB; *Tx* treatment.

To discuss actions needed for integration of WGS into routine TB diagnostics in the Kyrgyz Republic, a two-day workshop was held with 24 clinical, laboratory, and programmatic experts of nine national and international stakeholder organizations. The experts specified required data, plans, documents, and policies for incorporating the WGS technology into the national diagnostic algorithms (Table S4; Fig. 2). It was decided that WGS shall be used to rapidly determine the genomic resistance patterns of isolates diagnosed to be rifampicin-, MDR-, or poly-drug-resistant towards both isoniazid and fluoroquinolones.

## Discussion

Although gDST by WGS has advanced as a serious alternative to pDST^8,10,21–23^ its uptake for DR-TB diagnostics is currently hampered by severe concerns about the viability of its implementation in low- and middle-income countries^4^. Here, we present one example of WGS implementation that may serve as a blueprint for uptake in high TB prevalence settings. The six key lessons learnt from our project were, that (i) sequencing costs may be substantially higher than suggested by published estimates from the US; (ii) procurement and capacity building may take significantly longer than originally planned; (iii) the requirements for infrastructure need to be considered in an early project stage; (iv) quality assurance requires tailored solutions; (v) transitioning WGS to routine diagnostics demands careful planning; and (vi) ongoing support by experienced experts is required to ensure sustainable success.

Although costs of WGS have markedly dropped since its inception over ten years ago, initial material costs per sequence in Kyrgyzstan were almost twice as high as reported in other studies^12^. The high cost was partly caused by not using library preparation and sequencing kits to their full capacity during initial trainings, and partly by prices charged by the regional distributor exceeding those charged to laboratory counterparts in Europe and North America^12^. The budget estimates for WGS in high TB incidence countries need to be adjusted to the local situation based on reports from similar settings in order to avoid going over budget. The shipping and customs costs significantly impacted the overall budget because of the high frequency of shipments that were required due to the kits’ short guaranteed shelf-life of 3 months in average from the date of shipment which has also not changed for newer products like the ‘DNA Prep’ kits. Other companies offering library prep kits for Illumina sequencers like New England Biolabs (NEBNext Ultra II FS) or Qiagen (QIAseq FX) provide warranty periods of 6 months, however, when using them Illumina will refuse technical support whenever runs fail.

Another recurring cost is the yearly maintenance. We were quoted approximately twenty-six thousand USD per year for an all-inclusive-maintenance and warranty plan. To prevent a lapse in sequencing this needs to be brought in the TB program’s budget as early in advance as possible.

Our long implementation timeline was primarily caused by delays with importing large equipment due to national customs clearance policies. Those delays caused the postponement of trainings, which subsequently caused some of our reagents to expire unused. Sequencing kits should thus only be procured when the equipment is ready to be used, and not procured together with the equipment. Contracting a local customs clearance company helped prevent further delays at customs.

It is imperative that a local infrastructure is created meeting the requirements specified in the MiSeq Site Prep Guide with regards to both the room and information technology^20^. The sequencer shall be installed in a separate climatized room with access control, dust filtering, and sun-protection. The size of room and bench available may add restrictions to the instruments, for example, the Ion GeneStudio S5 sequencer (Ion Torrent ThermoFisher, Waltham, Massachusetts, USA) was alternatively considered but its footprint would not have fit in the available space^24^. For institutions aiming at advanced bioinformatic data analysis, a special WGS server with arbitrarily expandable storage capacities is recommended to tackle with the steadily increasing volume of data^25^. Because of budgetary restrictions, this was not possible during our project. The provided IT infrastructure only allows to analyse fastq-files for resistance and basic phylogeny but does not provide enough storage for cluster analysis of larger numbers of strains. When WGS will be routinely performed for both diagnostics and surveillance, a fully functional bioinformatic server infrastructure will need to be established. This will go along with six to ten times higher initial investment and with yearly maintenance costs which need to be allocated in the yearly budget.

ISO 15189 demands for yearly external quality assessment of every diagnostic test^26^. However, no external quality control is offered for WGS by an authorized organization such as Instand e.V. or EMQN. To verify the results, we performed an interlaboratory comparison. According to ISO 15189-2014-11 direct comparisons may substitute external quality controls. However, as more laboratories implement WGS, a standardized quality control system will be required^21^.

The integration of gDST into the national TB diagnostic algorithm demands regular policy updates^22^ because of the rapidly growing catalogues of mutations associated with resistance for the interpretation of genomic resistance profiles^10,23^. This requires a high level of autonomy and flexibility within the national board of experts accompanying and supervising gDST, and continuous technical support from international partners.

Current approaches to programmatic management of drug resistant TB in resource limited settings mainly rely on PCR based rapid tests for only very few genetic resistance markers or on pDST requiring biosafety-level 3 laboratory infrastructure which is rarely available or accessible^27–29^. As a consequence, TB-doctors initiate MDR-TB treatment mostly without essential information on the pathogen’s susceptibility. Next generation sequencing based technologies such as WGS offer the potential to rapidly and more comprehensively characterize the underlying resistome of TB bacteria^14^. However, a recent editorial came to the conclusion that this will “remain unavailable in tuberculosis endemic areas for the foreseeable future” and precision medicine for MDR-TB as part of routine practice consequently “remains a distant prospect in resource-limited settings, which carry the weight of global tuberculosis disease burden”^15^. We were able to provide the missing proof of concept and a template for the planning of the implementation of WGS in resource-limited settings which will hopefully help roll-out centers of expertise for rapid NGS based gDST particularly in all high MDR-TB burden countries^30^.

## Conclusions

Early initiation of effective treatment based on susceptibility patterns of the *Mtbc* pathogen is considered key to successful TB control in countries with high DR-TB incidence^4^. In addition to resistances to the established regimens, we are already witnessing an emergence of resistance to the newer DR-TB drugs such as bedaquiline. Recent advances of NGS, either by WGS of cultured *Mtbc* isolates or by tgNGS directly from clinical samples, offer fast and increasingly affordable high-resolution susceptibility profiles of TB bacteria^28^. Shared experience with successful implementation of WGS in high prevalence settings will facilitate the access to this technology in countries where it is most urgently needed.

## Supplementary Information

Supplementary Information.

## Data Availability

The datasets used and/or analyzed during the current study are available from the corresponding author on reasonable request.

## Abbreviations

DST: Drug susceptibility testing
DR-TB: Drug-resistant TB
XDR: Extensive drug resistant
EQA: External quality assessment
gDST: Genomic DST
HVAC: Heating, ventilation, and air conditioning
INH: Isoniazid
MDR: Multi-drug resistant
Mtbc: Mycobacterium tuberculosis complex
NGS: Next generation sequencing
NRL: National Reference Laboratory
pDST: Phenotypic DST
RIF: Rifampicin
SLID: Second-line injectable drug
SNP: Single nucleotide polymorphism
tgNGS: Targeted sequencing
TB: Tuberculosis
USAID: United States Agency for International Development
USD: United States Dollar
VAT: Value Added Tax
WGS: Whole genome sequencing
WHO: World Health Organization

## References

[1] Uplekar M (2015). WHO’s new end TB strategy. Lancet.

[2] WHO (2019). Global Tuberculosis Report 2019.

[3] Seung KJ, Keshavjee S, Rich ML (2015). Multidrug-resistant tuberculosis and extensively drug-resistant tuberculosis. Cold Spring Harb. Perspect. Med..

[4] Nahid, P. *et al*. Treatment of drug-resistant tuberculosis an official ATS/CDC/ERS/IDSA clinical practice guideline. *Am. J. Respir. Crit. Care Med.* **200**, e93–e142 (2019). 10.1164/rccm.201909-1874ST PMC6857485 31729908

[5] Shin SS (2012). Impact of rapid drug susceptibility testing for tuberculosis: program experience in Lima, Peru. Int. J. Tuberc. Lung Dis..

[6] Rodrigues C (2009). Drug susceptibility testing of Mycobacterium tuberculosis against second-line drugs using the BACTEC MGIT 960 System. Int. J. Tuberc. Lung Dis..

[7] Eliseev P (2016). The impact of a line probe assay based diagnostic algorithm on time to treatment initiation and treatment outcomes for multidrug resistant TB patients in Arkhangelsk Region, Russia. PLoS ONE.

[8] Gröschel MI (2018). Pathogen-based precision medicine for drug-resistant tuberculosis. PLoS Pathog..

[9] Feuerriegel S (2021). Rapid genomic first- and second-line drug resistance prediction from clinical Mycobacterium tuberculosis specimens using Deeplex-MycTB. Eur. Respir. J..

[10] Allix-Béguec C (2018). Prediction of susceptibility to first-line tuberculosis drugs by DNA sequencing. N. Engl. J. Med..

[11] WHO (2018). The Use of Next-Generation Sequencing Technologies for the Detection of Mutations Associated with Drug Resistance in *Mycobacterium tuberculosis* Complex: Technical Guide.

[12] Colman R (2019). Whole-genome and targeted sequencing of drug-resistant *Mycobacterium tuberculosis* on the iSeq100 and MiSeq: A performance, ease-of-use, and cost evaluation. PLOS Med..

[13] Helmy M, Awad M, Mosa KA (2016). Limited resources of genome sequencing in developing countries: Challenges and solutions. Appl. Transl. Genomics.

[14] Gordon AK, Marais B, Walker TM, Sintchenko V (2021). Clinical and public health utility of *Mycobacterium tuberculosis* whole genome sequencing. Int. J. Infect. Dis..

[15] Chakaya JM (2020). Programmatic versus personalised approaches to managing the global epidemic of multidrug-resistant tuberculosis. Lancet Respir. Med..

[16] The World Bank. The World Bank in the Kyrgyz Republic. https://www.worldbank.org/en/country/kyrgyzrepublic/overview (2020).

[17] WHO. Tuberculosis Profile: Kyrgyzstan. https://worldhealthorg.shinyapps.io/tb_profiles/?_inputs_&entity_type=%22country%22&lan=%22EN%22&iso2=%22KG%22 (2019).

[18] van Embden JD (1993). Strain identification of *Mycobacterium tuberculosis* by DNA fingerprinting: Recommendations for a standardized methodology. J. Clin. Microbiol..

[19] Kohl T (2018). MTBseq: A comprehensive pipeline for whole genome sequence analysis of *Mycobacterium tuberculosis* complex isolates. PeerJ.

[20] Illumina. MiSeq System Site Prep guide. https://emea.support.illumina.com/content/dam/illumina-support/documents/documentation/system_documentation/miseq/miseq-site-prep-guide-15027615-f.pdf (2014).

[21] Gargis AS (2012). Assuring the quality of next-generation sequencing in clinical laboratory practice. Nat. Biotechnol..

[22] Matthijs G (2016). Guidelines for diagnostic next-generation sequencing. Eur. J. Hum. Genet..

[23] Valafar SJ (2021). Systematic review of mutations associated with isoniazid resistance points to continuing evolution and subsequent evasion of molecular detection, and potential for emergence of multidrug resistance in clinical strains of *Mycobacterium tuberculosis*. Antimicrob. Agents Chemother..

[24] Quail MA (2012). A tale of three next generation sequencing platforms: comparison of Ion Torrent, Pacific Biosciences and Illumina MiSeq sequencers. BMC Genomics.

[25] Sboner A, Mu XJ, Greenbaum D, Auerbach RK, Gerstein MB (2011). The real cost of sequencing: Higher than you think!. Genome Biol..

[26] ISO. DIN EN ISO 15189:2014-11. Medical laboratories - Requirements for quality and competence (ISO 15189:2012, Corrected version 2014-08-15). Beuth Verlag GmbH (2014).

[27] Bernardo J, Yew WW (2009). How are we creating fluoroquinolone-resistant Tuberculosis?. Am. J. Respir. Crit. Care Med..

[28] Armstrong GL (2019). Pathogen genomics in public health. N. Engl. J. Med..

[29] Witney AA (2016). Clinical use of whole genome sequencing for *Mycobacterium tuberculosis*. BMC Med..

[30] WHO. WHO global lists of high burden countries for TB, multidrug/rifampicin-resistant TB (MDR/RR-TB) and TB/HIV, 2021–2025. Geneva: World Health Organization (2021).

[31] Merker M (2015). Evolutionary history and global spread of the *Mycobacterium tuberculosis* Beijing lineage. Nat. Genet..

