# Supplementary material for: Implementation of whole genome sequencing for tuberculosis diagnostics in a low-middle income, high MDR-TB burden country
Source: Sci Rep. 2021 Jul 28;11:15333. doi: 10.1038/s41598-021-94297-z (PMC8319420; doi:10.1038/s41598-021-94297-z)
Supplement: Supplementary file 1 — Supplementary Information. [file 41598_2021_94297_MOESM1_ESM.pdf]

Implementation of Whole Genome Sequencing for Tuberculosis diagnostics in a low-middle income, high MDR-TB burden country

Supplementary material

A. Supplementary Tables

Table S1: Computer specifications, locally built in Bishkek, Kyrgyzstan, as example for minimum requirements for NGS data management

Function	Part Name	Manufacturer	Quantity
Motherboard	Asus strix Z270H	Asus	1
Computer processing core	CPU Intel core i7-7700k	Intel	1
Memory modules	Adata DDR4 8 GB	Adata	8
Graphics card	VGA ASUS Dual GTX1060-6C Nvidia GeForce	Nvidia	1
Solid-State Drive	SSD Kingston 515 GB	Kingston	1
Hard-Drive	HDD 8 TB Toshiba 7200RPM	Toshiba	2
DVD drive	DVD drive	LG	1
Monitor	Monitor HikVision DS d5022QE	HikVision	1
External Hard-Drive	Adata 4 TB HDD	Adata	1
Software	MTBseq 1.0.3/1.0.4	RCB	1

Table S2: Costs of capacity building. Travel costs, training fees, number of training days, trainer and trainees, and objectives of training sessions required to successfully implement Next-Generation-Sequencing (NGS) in Bishkek, Kyrgyzstan

Training Objectives	Laboratory Location	Days	Number of Trainers	Number of Trainees	Travel costs (USD)	Estimated minimum training fees (USD)
DNA extraction in an established laboratory	IMLred, Gauting, Germany	4	1xSE; 1xTA	1xCM; 1xTA	$3,012.00	3,000.00
Basic technology of NGS in an established laboratory, NGS data analysis, library preparation, and machine use.	RCB, Borstel, Germany	16	1xSE; 1xPD; 1xTA	1xCM; 1xTA	$6,056.00	19,500.00
Proper use and maintenance of new on-site Fragment Analyzer 5200	NRL, Bishkek, Kyrgyzstan	3	1x AET	1xCM; 1xTA	*Included in purchase price*	
On-site DNA preparation on-site, laboratory technique, library preparation	NRL, Bishkek, Kyrgyzstan	10[a]	1xPD; 1xTA	1xCM; 1xTA	$2,780.00	6,250.00
Proper use and maintenance of new on-site MiSeq machine.	NRL, Bishkek, Kyrgyzstan	3	1x AEGT	1xCM; 1xTA	*Included in purchase price*	
First full on-site run of the NGS procedures with local equipment	NRL, Bishkek, Kyrgyzstan	5	1xSE	1xCM; 2xTA	$1,880.00	5,000.00
Refresher training, trouble-shooting, data management, NGS data analysis, Phylogenetic analysis; software training	NRL, Bishkek, Kyrgyzstan	12[a]	1xPD; 1xTA	1xCM; 2xTA	$2,976.00	7,500.00
**TOTAL**		**53**			**$16,704.00**	**$48,250.00**

Abbreviations: AET= Agilent Equipment Technician; AGET= Alliance Global Equipment Technician; CM=Clinical Microbiologist; IMLred= Institute of Microbiology and Laboratory Medicine; NGS= Next-Generation Sequencing; NRL= National Reference Laboratory; PD=Post Doc; SE= Senior Expert; TA=Technical Assistant;
[a]Split into 2 equal length training sessions.

Table S3: List of small equipment, consumables, reagents required for the implementation of Next Generation Sequencing in Bishkek, Kyrgyzstan

Category	Item Description	Manufacturer	Distributor	Catalogue Number	Qty (units)	Unit	Price/unit (USD)	Sub-Total (USD)
Reagents	Tween 20	Atlas Chemical Industries	VWR	437082Q	8	100 ml	36.48	291.84
	Tris (hydroxymethyl) aminomethane hydrochloride (Tris hydrochloride) (ULTRA PURE GRADE)	Amresco	Vizamed	0234-1.000	1	1 kg	297.00	297.00
	Ethylenediaminetetraacetic acid EDTA (EDTA). (ULTRA PURE GRADE)	Amresco	Vizamed	0322-1.0	1	1 kg	107.00	107.00
	Lysozyme from egg white (ULTRA PURE GRADE)	Amresco	Vizamed	0663-0.005	1	5 gr	192.00	192.00
	Sodium dodecyl sulfate (Sodium lauryl sulfate) (BioChemica)	Applichem	Vizamed	A2572.1000	1	1 kg	460.00	460.00
	Proteinase K (BIOCHEMISTRY)	BioChemistry	Vizamed	1.204568.0	2	100 mg	330.00	660.00
	Sodium chloride (sodium chloride) (EXTRA PURE)	Local Producer	Vizamed	215	2	500 gr	6.00	12.00
	Cetyltrimethylammonium bromide (CTAB) (HIGH PURITY GRADE)	Amresco	Vizamed	0833-1.000	1	1 kg	417.00	417.00
	Chloroform (Trichloromethane) (BIOTECHNOLOGY GRADE)	Amresco	Vizamed	0757-0.950	3	950 ml	173.00	519.00
	Alcohol isoamyl (2-methylbutanol-4) (Molecular biology grade)	Applichem	Vizamed	A2610.0500	1	500 ml	238.00	238.00
	Isopropyl alcohol (2-propanol) (BIOTECHNOLOGY GRADE)	Amresco	Vizamed	0918-1.000	1	1000 ml	105.00	105.00
	Isopropyl alcohol (2-propanol)	Amresco	Vizamed	0918-500	2	500 ml	53.00	106.00
	MagSi-NGS Prep Plus (Magnetic beads)	Omega Bio-tek	VWR	M1378-01	1	50 ml	604.20	604.20
	Molecular Biology Grade purified water	VWR	VWR		3	1000 ml	46.51	139.53
Sub-total regents					*28*			*4,148.57*
Small equipment	Automated Pipette 0.5-10 µl	Eppendorf	NeoLab	E-0946	3	1 pc	189.92	569.76
	Automated Pipette 10-100 µl	Eppendorf	NeoLab	E-0947	2	1 pc	189.92	379.84
	Automated Pipette 20-200 µl	Eppendorf	NeoLab	E-1865	1	1 pc	289.56	289.56
	Automated Pipette 100-1000 µl	Eppendorf	NeoLab	E-0948	2	1 pc	189.92	379.84
	Autopipette E3	Eppendorf	NeoLab	E-9105	1	1 pc	652.16	652.16
	Autopipette M4	Eppendorf	NeoLab	4982000411	1	1 pc	203.01	203.01
	Vortex	SunLab	NeoLab	D-9800	1	1 pc	119.07	119.07
	Plate shaker for 2 plates	NeoLab	NeoLab	7-0055	1	1 pc	1.072.68	1.072.68
	Micro plate centrifuge	Axygen	NeoLab	C-2049	1	1 pc	553.87	553.87
	Compact table centrifuge 5702 Eppendorf	Eppendorf	NeoLab	E-1901	1	1 pc	1.596.00	1.596.00

	Swinging rotor with 4 round cups - Model A-4-38	Eppendorf	NeoLab	5702720003	1	1 pc	837.90	837.90
	Adaptor for 2.6-7mL tubes	Eppendorf	NeoLab	5702719005	2	1 pc	282.72	565.44
	Adaptor for 1.5-2mL tubes	Eppendorf	NeoLab	5702745006	2	1 pc	282.72	565.44
	Adapter for 85mL round cups	Eppendorf	NeoLab	5702735000	2	1 pc	282.72	565.44
Sub-total small equipment					*21*			*8,350.00*
Reusable items	Rack	NeoLab	NeoLab	2-1837	5	1 pc	11.63	58.15
	CellCamper Cryo-rack for 96 PCR tubes	NeoLab	NeoLab	2-3733	2	1 pc	99.09	198.18
	Magnetic rack (DynaMag PCR)	Thermofisher	Thermo Fisher	492025	1	1 pc	405.84	405.84
	Cryobox - Cardboard	VWR	VWR	479-1417	75	1 pc	2.51	188.25
Sub-total items					*83*		*519.06*	*850.42*
Non-reusable items	epDualfilter T.I.P.S. sterile Tips 1-10µl	Eppendorf	NeoLab	E-6521	2	100 pcs	66.67	133.34
	epDualfilter T.I.P.S. sterile Tips 0.5-20µl	Eppendorf	NeoLab	E-6510	33	960 pcs	142.50	4.702.50
	epDualfilter T.I.P.S. sterile Tips 2-100µl	Eppendorf	NeoLab	E-6511	30	960 pcs	142.56	4.276.80
	epDualfilter T.I.P.S. sterile Tips 2-200µl	Eppendorf	NeoLab	E-6511	10	960 pcs	461.70	4.617.00
	epDualfilter T.I.P.S. sterile Tips 50-1000µl	Eppendorf	NeoLab	E-6513	39	960 pcs	159.89	6.235.71
	epDualfilter T.I.P.S. standard Tips 2-200µl	Eppendorf	NeoLab	E-6511	3	960 pcs	461.70	1.385.10
	epDualfilter T.I.P.S. standard Tips 50-1000µl	Eppendorf	NeoLab	E-6513	4	1000 pcs	66.84	267.36
	NeoLab combitips 5mL	Eppendorf	NeoLab	E-6405	4	100 pcs	82.55	330.20
	NeoLab combitips 2.5mL	Eppendorf	NeoLab	106989650	1	100 pcs	152.26	152.26
	Serological pipette tips PE	Eppendorf	NeoLab	0030127722	1	400 pcs	82.55	82.55
	Pasteur pipette. 3 ml	RatioLab	NeoLab	1-6157	4	500 pcs	28.80	115.20
	PCR 8 strip vials with flat lids	VWR	VWR	732-1164	4	125 pcs	139.08	556.32
	PCR vials 0.5mL	Eco-Lab	NeoLab	E-1030	3	1000 pcs	23.94	71.82
	Screw cap Cryo-Vial 2mL	Simport	NeoLab	7-8051	25	100 pcs	36.25	906.25
	Safelock vials 2mL sterile	Eppendorf	NeoLab	E-2315	25	100 pcs	37.73	943.25
	Centrifugation tube 50ml	MoonLab	NeoLab	4-0003	1	500 pcs	67.26	67.26
	96 Well plate	TwinTec	VWR	732-0106	1	25 plates	210.90	210.90
	X50 1mL DeepWell plate	FisherBrand	ThermoFisher	1138155	1	50 plates	125.63	125.63
	Laboratory glass - 5000mL	Kimax	NeoLab	1-0200	2	1 pc	56.09	112.18
	Laboratory glass-1000mL	Kimax	NeoLab	1-0198	5	1 pc	5.99	29.95
	Laboratory glass-500mL	Kimax	NeoLab	1-0197	5	1 pc	3.74	18.70
	Laboratory glass-250mL	Kimax	NeoLab	1-0197	10	1 pc	2.15	21.50
	Laboratory glass - 100mL	Kimax	NeoLab	1-0195	10	1 pc	1.88	18.80

Inoculating loops	MoonLab	NeoLab	4-0055	2	500 pcs	19.95	39.90
Cotton Swabs	NeoLab	NeoLab	2-1019	10	100 pcs	8.05	80.50
Microseal film	VWR	VWR	210-0005	1	100 pcs	80.36	80.36
Nitrile Gloves	NeoProtect	NeoLab	2264871	6	1 box	5.99	35.94
Surgical Mask	Kimberly-Clark	111-9004	VWR	1	50 pcs	23.14	23.14
KimTech Absorbent paper	Kimtech	VWR	115-2075	1	15 boxes	105.45	105.45
Round stickers	VWR	8175004	VWR	1	5000 pcs	228.00	228.00
Paint Marker	Hahnmuhle	Neolab	2-1968	2	1 pc	9.63	19.26
Sub-total non-reusable items				*247*			*25,993.13*
**Total**				**379**			**$ 39,342.13**

Table S4: Transitioning planning and integration of NGS in routine TB diagnostic algorithm in Kygyzstan. Required documents, policies and orders

**NGS documents, plans, and strategies to be prepared, endorsed, implemented, and monitored**
Validation of NGS technologies (WGS, tgNGS[1]) for diagnostics of *M. tuberculosis* resistances
Establishment of internal and external Quality Assurance measures
Registration of Illumina MiSeq equipment and reagents by Kyrgyz custom
Endorsement of NGS technology for TB diagnostics by Kyrgyz MoH and NTP
Strategy for improved sample and report logistics
Revision of National Laboratory Strategic Plan to consider NGS technology rollout
Revision of national diagnostic algorithms and guidelines
Budget calculation and forecasting for three to five years considering reagents, consumables, maintenance, staffing
Maintenance plan
National Drug Resistance Survey using NGS technologies

1 WGS, whole genome sequencing; tgNGS, targeted next generation sequencing

Protocol (standard operation procedure) for DNA extraction used in this project:

**Standard Operating Procedure**		
**Isolation of genomic DNA from *Mycobacterium tuberculosis***		
**STTH_SOP_M_010.01**		Das Bildelement mit der Beziehungs-ID rId7 wurde in der Datei nicht gefunden.
**Project area**	KGZ NRL	
Application area	Molecular Department	
Replaced version from	New version	
**Entry into force**	**Annulated**	
**Forms**	*F1. Form StopTTH_F_001_DNAEx_Extraction and Control of DNA.*	
**Keywords**		
**Laboratory area**		Number of copies
	**Compiled by**	**Approved by**
Date		
Signature		
Name		

CONTENTS

1. OBJECTIVES & SCOPE 9
2. ABBREVIATIONS AND DEFINITIONS 9
3. DESCRIPTION 9
4. TASKS, RESPONSIBILITIES 12
5. RELATED DOCUMENTS 12
6. RELATED FORMS 12
7. REFERENCES 12
8. LIST OF CHANGES 12
9. ACKNOWLEDGEMENT OF UNDERSTANDING 12

1. Objectives & Scope

1.1. This SOP describes the procedure for DNA Extraction for further using in WGS.

2. Abbreviations and definitions

**Abbreviation**	**Definition**
BSC	Biosafety Cabinet
DNA	Deoxyribonucleic acid
LJ	Lowenstein-Jensen
N/A	Not applicable
RT	Room temperature
SOP	Standard Operational Procedure
SNPs	Single nucleotide polymorphisms
STOP TTH	Stop transmission of drug resistant tuberculosis in Central Asian TB hospitals
WGS	Whole-genome sequencing

3. Description

3.1. Reagents, consumable, and equipment

Product	***Suggested Company***	***Catalog number***
**Equipment**		
BSC, Class II		
Water bath		
Incubator		
Centrifuge R		
Centrifuge spin		
Refrigerator		
Freezer -20C°		
Thermoblock		
**Small Equipment**		
Pipet 1-10 µl		
Pipet 10-100 µl		
Pipet 100-1000 µl		
Timer		
Vortex		
Thermometer for water		
**Reagents**		
TE buffer	Laboratory prepared	
Lysozyme 10 mg/ml	Laboratory prepared	
Mix SDS/Proteinase K	Laboratory prepared	
Mix CTAB/NaCl	Laboratory prepared	
NaCl 5M	Laboratory prepared	
Mix Chloroform/Isoamyl alcohol	Laboratory prepared	

Isopropanol	Laboratory prepared	
Ethanol 70% cold,	Laboratory prepared	
Tris/HCl-Buffer	Laboratory prepared	
**Reusable Consumables**		
Rack		
Discarded box		
Cold box		
**Non Reusable Consumables**		
Tips 1-10 µl		
Tips 10-100 µl		
Tips 100-200 µl		
Tips 100-1000 µl		
Micro centrifuge tube, 2 ml		
Lid for micro centrifuge tubes		
Gloves		
Loop		
Absorbent paper		

3.2. Principle

3.2.1. Whole-genome sequencing (WGS) is a method established as the potential as a diagnostic test to identify species and as many drug resistance-conferring mutations.

3.2.2. This method might be defined as a high-resolution method of linking cases to outbreaks by identifying single nucleotide polymorphisms (SNPs).

3.2.3. WGS can produce results faster than current culture-based methods, thus this method is already used routinely in a number of clinical and public health laboratories worldwide.

3.2.4. Isolation of high quantities of pure, intact, double stranded, highly concentrated, not contaminated genomic DNA is prerequisite for successful and reliable genotyping analysis.

3.2.5. This SOP describes the steps for DNA extraction using for Genome Sequencing with MiSeq methods.

3.3. Samples

3.3.1. Positive strains grown on LJ culture medium.

3.3.1.1. Grow strains of interest on Lowenstein-Jensen medium at 37°C until growth becomes clearly visible.

!!! NOTE. Handling with positive strains should be done in BSL 2 Laboratory, using BSC class II, verified and certified annually.

3.3.2. Procedure

3.3.2.1. Perform the Procedure in BSC, class II.

3.3.2.2. Prepare and label sufficient number of micro centrifuge tube (according to the samples numbers).

3.3.2.3. Add 400 µl TE-Buffer in each micro centrifuge tubes.

3.3.2.4. Transfer an appropriate number (1-3 inoculation loops) of bacterial cells into a tube containing 400 µl TE-Buffer.

3.3.2.5. Incubate for 20 min at 80°C in a water bath to kill bacteria.

- *Check temperature with thermometer as this seems to be crucial for DNA quantity and quality afterwards.*

3.3.2.6. Add 50 µl of 10mg/ml Lysozyme.
3.3.2.7. Vortex shortly the tubes.
3.3.2.8. Incubate at least 1 h at 37 °C or overnight (best o/n!).
3.3.2.9. Add 75 µl of 10% SDS/Proteinase K mix (5µl Proteinase K (10 mg/ml) + 70 µl 10% SDS).
3.3.2.10. Vortex shortly.
3.3.2.11. Incubate 10 min at 65 °C in the water bath.
3.3.2.12. Add 100 mkl CTAB/NaCl mix (pre warmed at 65°C).

- Can be introduced with samples in previous incubation.

3.3.2.13. Add 100 µl 5M NaCl.
3.3.2.14. Vortex until the liquid content becomes white.
3.3.2.15. Incubate 10 min at 65° C in the water bath.
3.3.2.16. Add approx. 750 µl chloroform/isoamyl alcohol mix (24:1).
3.3.2.17. Vortex 10 sec.
3.3.2.18. Centrifuge 15 min at 13000 rcf at RT.
3.3.2.19. Transfer aqueous supernatant in a new sterile micro centrifuge tube.

- **Be careful**. Do not take the white pellets. In case if white pellets was taken, discard supernatants in original tube and centrifuge it again (15 min, 13000 rcf at RT).

3.3.2.20. Add 0,6volume (450 mkl) isopropanol. Close the lid.
3.3.2.21. Mix carefully (invert tube several times.
3.3.2.22. Incubate 30 min at -20°C ().

- Can be incubated more than 30 min.

3.3.2.23. Centrifuge 15 min at 13000 g.
3.3.2.24. Remove most of supernatant direct form the tube. Dry the edges of tubes on the absorbent paper.
3.3.2.25. Add 500 mkl of cold 70% ethanol.
3.3.2.26. Add a new sterile lid to each sample tube.
3.3.2.27. Invert 2 times tubes.
3.3.2.28. Centrifuge 5 min at 13000 g.
3.3.2.29. Discard supernatant.
3.3.2.30. Centrifuge 5 min at 13000 g.
3.3.2.31. Discard cautiously the last µl of supernatant using tips 2-10 mkl.

- **Be careful**. Do not take the sediments.

3.3.2.32. Discard lids.
3.3.2.33. Dry pellet about 20-30 min inside of BSC (without lids).
3.3.2.34. Add 80 µl TrisHCl buffer without EDTA (or pure water).
3.3.2.35. Incubate DNA for 20 min at 60°C (for DNA dissolving).
3.3.2.36. Control quality and concentration of the DNA on DeNovix and next on Qubit.
3.3.2.37. Store DNA samples at -20°C.

4. Tasks, responsibilities

Task	Responsible
Subculture on LJ tube	StopTTH Lab Technician
Extraction of pure DNA	StopTTH Lab Technician
Storage of DNA	StopTTH Lab Technician
Supervising the process and validation of the results	StopTTH Lab Doctor

5. Related Documents

5.1.1. N/A.

6. Related Forms

6.1.1. *Form StopTTH-SOP-M010-F-001-DNAEx-Extraction and Control of DNA.*

7. References

7.1.1. Instruction from Molecular Laboratories, Borstel.

8. List of Changes

Version	Changes	Come into force
20180426	New	20180426

9. Acknowledgement of understanding

By signing and dating below, I acknowledge that I have read and understood the SOP.

Date	Name	Position	Signature

C Supplementary Figures

Legends to supplementary figures:

Figure S1 (page 14)**:** Gantt Chart of NGS implementation in Kyrgyzstan. The chart reflects the real time required for the respective project phases. Administrative and legal framework was set-up after 16 weeks. Procurement of equipment and materials required 46 weeks from planning of needs until on-site installation. Capacity building was divided in seven training units. The Kyrgyz master users were able to autonomously perform NGS after the sixth training. External Quality Assessment was completed in project week. 69. Two weeks later transitioning of NGS to the national TB program was prepared in a workshop with national and international stakeholders.

Figure S2 (page 15)**:** Detailed analysis of one of the divergent samples: Both, RCB and Kyrgyz NRL identified wild-type and an SNP population each with a proportion of approximately 50% at the divergent position. Due to the inherent technical variation in library preparation and sequencing technology, the SNP was sequenced with a frequency of 52% at the RCB (upper half of the figure) which reported the allele, while the NRL (lower half of the figure) identified the wildtype at the same position in 57% of reads and consequently reported the wildtype.

Figure S1

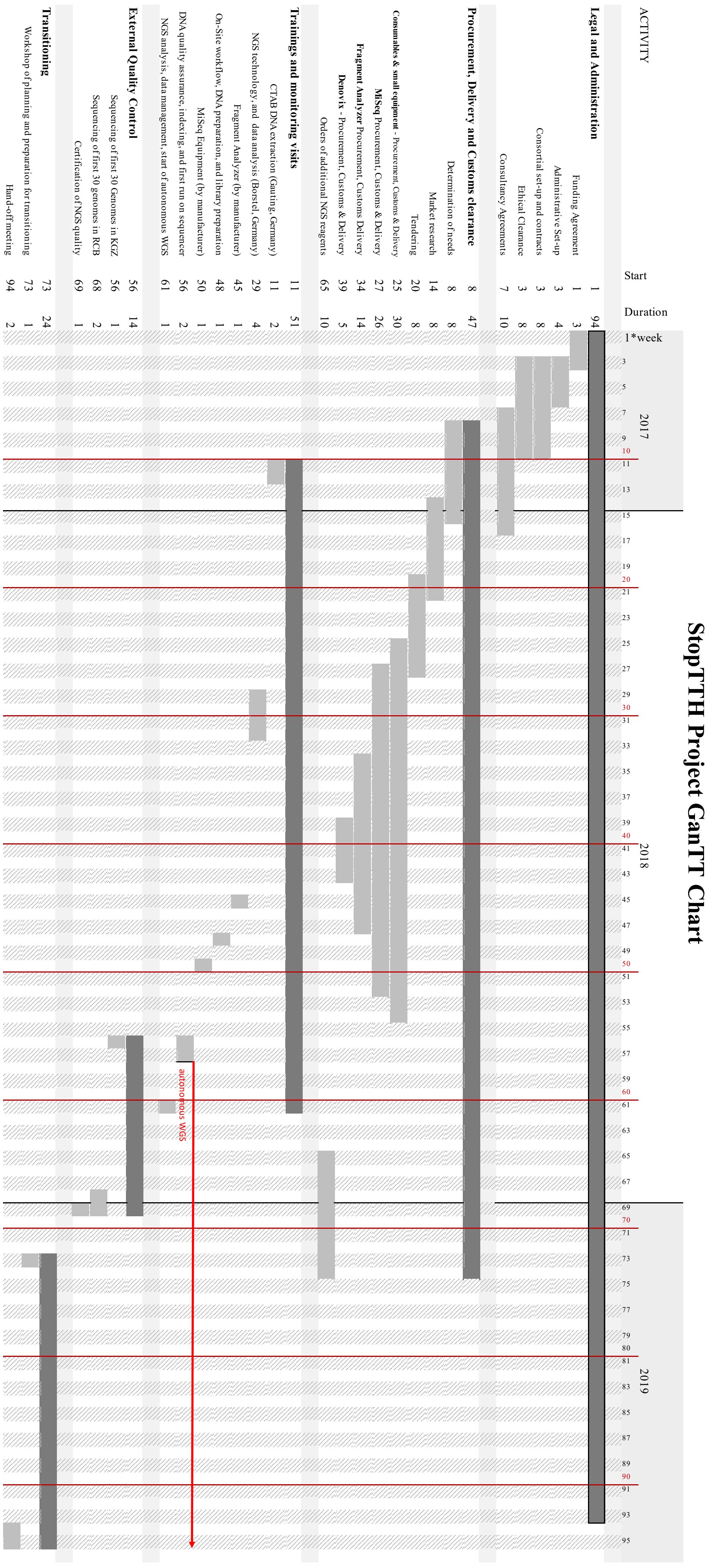
StopTTH Project GanTT Chart
ACTIVITY
Start
Duration
1*week
2017
2018
2019
Legal and Administration 1 94
Funding Agreement 1 3
Administrative Set-up 3 4
Consortial set-up and contracts 3 8
Ethical Clearance 3 8
Consultancy Agreements 7 10
Procurement, Delivery and Customs clearance 8 47
Determination of needs 8 8
Market research 14 8
Tendering 20 8
Consumables & small equipment - Procurement, Customs & Delivery 25 30
MiSeq Procurement, Customs & Delivery 27 26
Fragment Analyzer Procurement, Customs Delivery 34 14
Denovix - Procurement, Customs & Delivery 39 5
Orders of additional NGS reagents 65 10
Trainings and monitoring visits 11 51
CTAB DNA extraction (Gauting, Germany) 11 2
NGS technology, and data analysis (Borstel, Germany) 29 4
Fragment Analyzer (by manufacturer) 45 1
On-Site workflow, DNA preparation, and library preparation 48 1
MiSeq Equipment (by manufacturer) 50 1
DNA quality assurance, indexing, and first run on sequencer 56 2
NGS analysis, data management, start of autonomous WGS 61 1
External Quality Control 56 14
Sequencing of first 30 Genomes in KGZ 56 1
Sequencing of first 30 genomes in RCB 68 2
Certification of NGS quality 69 1
Transitioning 73 24
Workshop of planing and preparation for transitioning 73 1
Hand-off meeting 94 2
autonomous WGS

Figure S2:

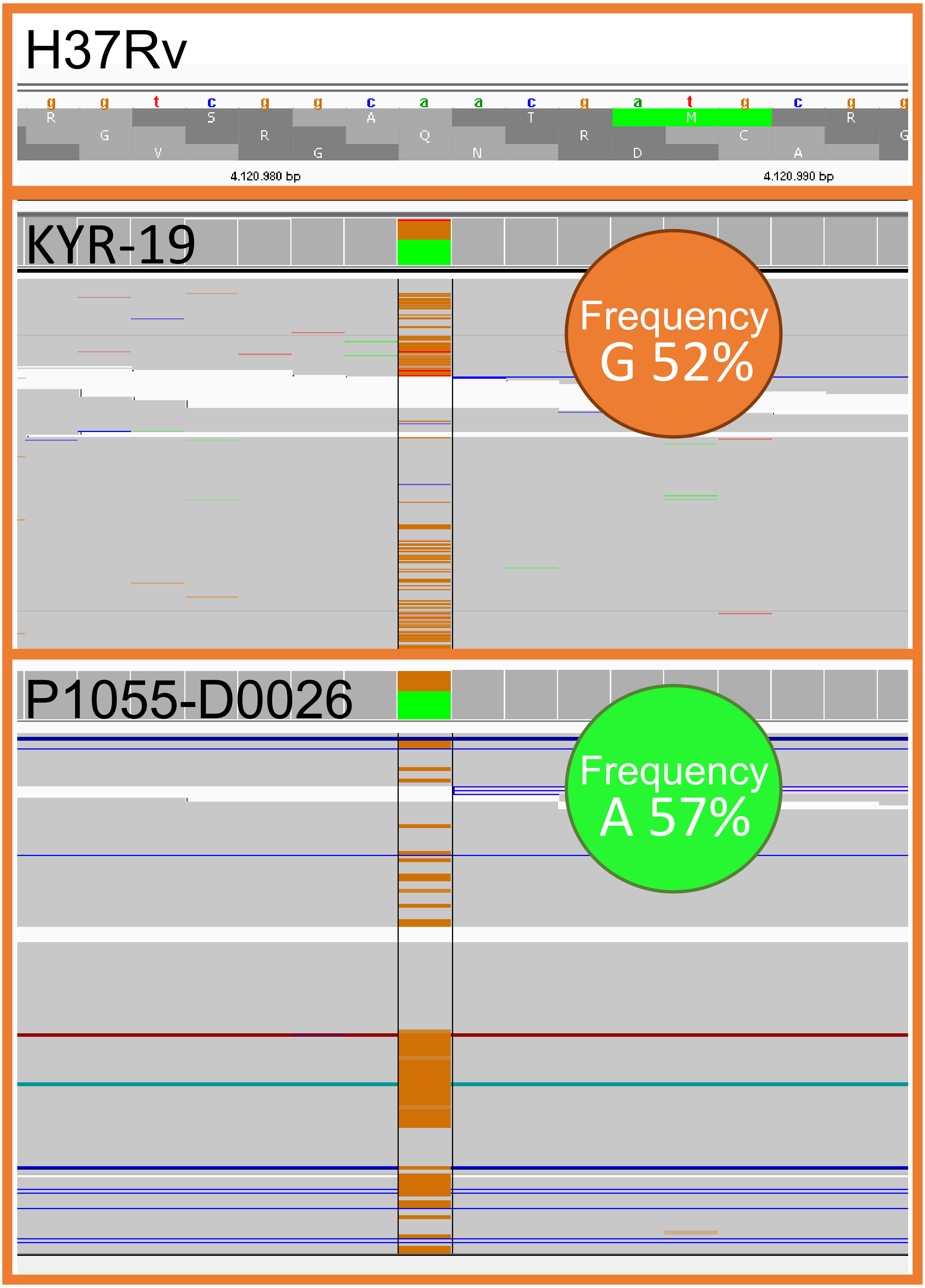
H37Rv
g g t c g g c a a c g a t g c g g
R S A T M R
G R Q R C G
V G N D A
4.120.980 bp
4.120.990 bp
KYR-19
Frequency
G 52%
P1055-D0026
Frequency
A 57%
